# Reply: Guideline recommendations don't exist in a vacuum

**DOI:** 10.1183/13993003.01421-2024

**Published:** 2024-09-26

**Authors:** Steven Walker, Rob Hallifax, Najib Rahman, Nick Maskell

**Affiliations:** 1 Academic Respiratory Unit, Southmead Hospital, Bristol, UK; 2 North Bristol Lung Centre, Southmead Hospital, Bristol, UK; 3 Oxford Centre for Respiratory Medicine, Oxford University Hospitals NHS Trust, Oxford, UK; 4 Oxford Respiratory Trials Unit, University of Oxford, Oxford, UK; 5 Oxford Chinese Academy of Medicine Institute, Oxford, UK

## Abstract

The letter “Debunking the myth: why wall suction should not be routine in pneumothorax treatment” postulated that negative pressure *via* thoracic suction may lead to worse outcomes for patients and suggests that the joint ERS/EACTS/ESTS clinical practice guidelines on adults with spontaneous pneumothorax [1] should be amended.

## Shareable abstract

**ERS guidelines must be evidence led. Until this evidence exists, recommendations cannot be made for or against an intervention, such as suction, where the relevant evidence base is very poor.** https://bit.ly/4dFXBDR

*Reply to I.S. Tournoy and K.G. Tournoy:*

The letter “Debunking the myth: why wall suction should not be routine in pneumothorax treatment” postulated that negative pressure *via* thoracic suction may lead to worse outcomes for patients and suggests that the joint ERS/EACTS/ESTS clinical practice guidelines on adults with spontaneous pneumothorax [1] should be amended.

This hypothesis may be correct. It could also be wrong.

There is little comparative data comparing immediate thoracic suction to no suction in patients with spontaneous pneumothorax (SP). One randomised trial, completed more than 40 years ago, found no difference in lung re-expansion rates or hospital stay [2]. Another under-powered randomised study of initial suction in 29 patients found no difference in success rates [3]. We have little confidence that either study design would demonstrate a treatment effect either way, if one existed.

I.S. Tournoy and K.G. Tournoy argue that the lung should re-expand with a chest tube, without assistance of negative pressure. We agree: almost all spontaneous pneumothoraces will resolve with time. However, this time can be substantial, as observational studies have shown [4]. In such cases, the premise that suction will induce pleural apposition and seal the pleural defect is attractive. It is worth noting that the guideline question specifies that its recommendation is for patients with persistent air leak [1].

The authors argue that suction leads to complications. We agree, the high-pressure, high-volume suction approach they dispute should not be used. All suction systems should be high-volume, low-pressure (not high-pressure), for the reasons outlined. The cited literature reviewed from 1988 is not the correct way to prove that suction induces re-expansion pulmonary oedema [5].

We welcome the debate; however, the joint ERS/EACTS/ESTS guideline, using the GRADE (Grading of Recommendations, Assessment, Development and Evaluations) approach, must be evidence-led. Until this evidence exists, recommendations cannot be made for or against an intervention, such as suction, where the relevant evidence base is very poor.

We need not wait long: the currently recruiting Randomised trial of Suction for Primary Pneumothorax Early Resolution study (www.isrctn.com/ISRCTN18017504) is comparing early use of thoracic suction to no suction in patients with primary spontaneous pneumothorax, and we hope will provide definitive evidence of safety and clinical effectiveness.

## Shareable PDF

10.1183/13993003.01421-2024.Shareable1 This one-page PDF can be shared freely online.
Shareable PDF ERJ-01421-2024.Shareable
