# Supplementary material for: Reply: Guideline recommendations don't exist in a vacuum
Source: Eur Respir J. 2024 Sep 26;64(3):2401421. doi: 10.1183/13993003.01421-2024 (PMC11424924; doi:10.1183/13993003.01421-2024)
Supplement: Supplementary file 1 [file ERJ-01421-2024.Shareable.pdf]

SHAREABLE PDF

Reply: Guideline recommendations don't exist in a vacuum

Steven Walker[1,2], Rob Hallifax[3,4], Najib Rahman[3,4,5] and Nick Maskell[1,2]

[1]Academic Respiratory Unit, Southmead Hospital, Bristol, UK. [2]North Bristol Lung Centre, Southmead Hospital, Bristol, UK. [3]Oxford Centre for Respiratory Medicine, Oxford University Hospitals NHS Trust, Oxford, UK. [4]Oxford Respiratory Trials Unit, University of Oxford, Oxford, UK. [5]Oxford Chinese Academy of Medicine Institute, Oxford, UK.

Corresponding author: Steven Walker (steven.walker@bristol.ac.uk)

Shareable abstract (@ERSpublications)
ERS guidelines must be evidence led. Until this evidence exists, recommendations cannot be made for or against an intervention, such as suction, where the relevant evidence base is very poor. https://bit.ly/4dFXBDR

Cite this article as: Walker S, Hallifax R, Rahman N, *et al*. Reply: Guideline recommendations don't exist in a vacuum. *Eur Respir J* 2024; 64: 2401421 [DOI: 10.1183/13993003.01421-2024].

This extracted version can be shared freely online.

Copyright ©The authors 2024.

This version is distributed under the terms of the Creative Commons Attribution Licence 4.0.

Received: 5 Aug 2024
Accepted: 7 Aug 2024

Reply to I.S. Tournoy and K.G. Tournoy:

The letter "Debunking the myth: why wall suction should not be routine in pneumothorax treatment" postulated that negative pressure *via* thoracic suction may lead to worse outcomes for patients and suggests that the joint ERS/EACTS/ESTS clinical practice guidelines on adults with spontaneous pneumothorax [1] should be amended.
